# Study protocol: individualized music for people with dementia - improvement of quality of life and social participation for people with dementia in institutional care

**DOI:** 10.1186/s12877-018-1000-3

**Published:** 2018-12-14

**Authors:** Lisette Weise, Elisabeth Jakob, Nils Frithjof Töpfer, Gabriele Wilz

**Affiliations:** Department of Counseling and Clinical Intervention, Institute of Psychology, Friedrich Schiller University of Jena, Humboldtstrasse 11, 07743 Jena, Germany

**Keywords:** Dementia, Alzheimer’s disease, Non-pharmacological intervention, Individualized music, Receptive music therapy, Behavioral and psychological symptoms of dementia, Quality of life, Problem behavior, Institutional care, Long-term care

## Abstract

**Background:**

People with dementia (PwD) experience a range of negative behavioral and psychological symptoms which can lower their quality of life. Because of the increasing prevalence of dementia, interventions that maintain and enhance the quality of life for PwD are needed. Listening to individualized music constitutes a promising non-pharmacological intervention for PwD. However, despite some preliminary results, evidence regarding the effectiveness of such interventions has been mixed and previous studies have shown a number of methodological limitations. In a randomized controlled trial, we address the limitations of previous research and assess the feasibility, efficacy, and acceptability of an individualized music intervention for PwD living in a nursing home.

**Methods:**

Residents with dementia from four to five nursing homes in Germany will be randomly assigned to either an intervention or control group. The intervention group will listen to personally-relevant music for 20 min every other day for six weeks. Nursing staff will assess participants’ quality of life and problem behavior at the six-week baseline, pretest, posttest, and at the six-week follow-up. Additionally, the participants’ behavior will be observed during the intervention period by project staff. The implementation, acceptance, and applicability of the intervention will also be evaluated.

**Discussion:**

The study results will show whether an individualized music intervention can improve the quality of life for PwD living in a nursing home. Additionally, it will provide valuable insight into the acceptability and implementation of an individualized music intervention in the institutional care setting. If the individualized music intervention proves to be effective and widely applicable, it could be implemented on a large scale in institutional care as an easy-to-administer intervention.

**Trial registration:**

German Clinical Trials Register DRKS00013793; ISRCTN registry, ISRCTN59052178, date applied 27 February 2018, date assigned 4 April 2018, retrospectively registered.

## Background

The increasing prevalence of dementia raises the question of how the quality of life and care for people with dementia (PwD) can be maintained and enhanced. PwD experience a range of negative behavioral and psychological symptoms such as agitation and depression [1, 2] which can lower not only their own quality of life but also that of their caregivers [3–5]. To avoid potential negative side-effects of pharmacological therapies [6–8], it is widely suggested that non-pharmacological interventions, which help create more satisfying living environments for PwD, are desirable [9, 10]. In this regard, PwD are thought to particularly benefit from individualized music interventions based on an individual’s own preferences and experiences.

Previous research shows that listening to personally meaningful music can facilitate positive social interactions [11] and promote positive emotions and memories as well as a reduction of stress, agitation, and anxiety in PwD [12–14]. The potential benefit of individualized music interventions for PwD is also discussed in the neuroscience literature, as dementia affects the specific brain areas (supplementary motor area) associated with long-term music memory functions to a lesser extent [15, 16]. Therefore, PwD continue to remember personally-relevant music very well. Moreover, listening to individualized music initiates a complex interaction between different brain regions which results in alertness, relaxation, interest, and a positive mood [17].

Thus, individualized music can be regarded as a promising non-pharmacological intervention for PwD to improve their quality of life. Quality of life is understood here as a superordinate construct which refers to an improvement of well-being and social participation as well as a reduction of depressive symptoms and problem behavior.

Preliminary evidence with regard to the effectiveness of individualized music interventions has been provided for the reduction of agitation and anxiety [18–20] as well as for increases in communication behavior, well-being, and the expression of positive emotions [21, 22]. Nevertheless, the amount of studies is limited and previous evaluations showed several methodological limitations, as most of them were quasi-experimental and pilot-trials including only small samples. Evidence about the effectiveness of music interventions in general (active and passive form) has been mixed [23]. In addition to the mostly poor methodological quality of studies which has been noted by systematic reviews and meta-analyses [23–28], interpreting the results of previous music intervention evaluations is difficult due to the diversity of intervention approaches (e.g. with regard to dosage). In particular, previous evaluations have seldom reported precisely how the intervention was implemented (e.g. how personally-relevant music was identified) or assessed implementation success.

In sum, although previous reviews and individual studies have provided some evidence that individualized music interventions can improve the quality of life for PwD, evidence regarding the effects of individualized music interventions for PwD is mixed and more methodologically rigorous studies are needed.

In the current study, we address the methodological limitations of previous studies by using a randomized controlled trial (RCT) with a large sample size, following a careful implementation procedure and evaluating the implementation success. The aim of our study is to assess the feasibility, efficacy, and acceptability of an individualized music intervention for PwD in institutional care. The primary objective is to evaluate whether the individualized music intervention improves the quality of life of PwD compared to standard care. Further objectives are to evaluate whether the individualized music intervention can be implemented successfully in the institutional care setting and to determine the level of acceptability in relation to the intervention.

We hypothesize an improvement of quality of life in participants in the intervention group (IG) compared to the control group (CG). In particular, the intervention is expected to enhance well-being, quality of sleep, and social participation as well as reduce depressive symptoms and problem behavior (i.e. agitation and resistance to care). In addition, we expect to observe positive short-term effects during and after the music listening sessions including emotional and motor changes (e.g. facial expression, body movement, and other positive reactions) as well as improvements in communication, attention, and orientation. We expect to find high levels of acceptability and that implementing the individualized music intervention in multiple nursing homes will be perceived as feasible.

## Methods

### Design

This study is a multi-center RCT with a baseline (T0; six weeks before treatment), pretest (T1), posttest (T2), and follow-up (T3; six weeks after treatment) design. The study will take place successively in four to five nursing homes in Thuringia, Germany. The participants will be randomly assigned to an intervention or a control group. Following T0, random allocation in a 1:1 ratio will be performed stratified by gender using a computer-generated randomization list for each nursing home. The randomization will be performed by a member of the study team not involved in the baseline assessment using the random number generator Random.org.

**Fig. 1:**
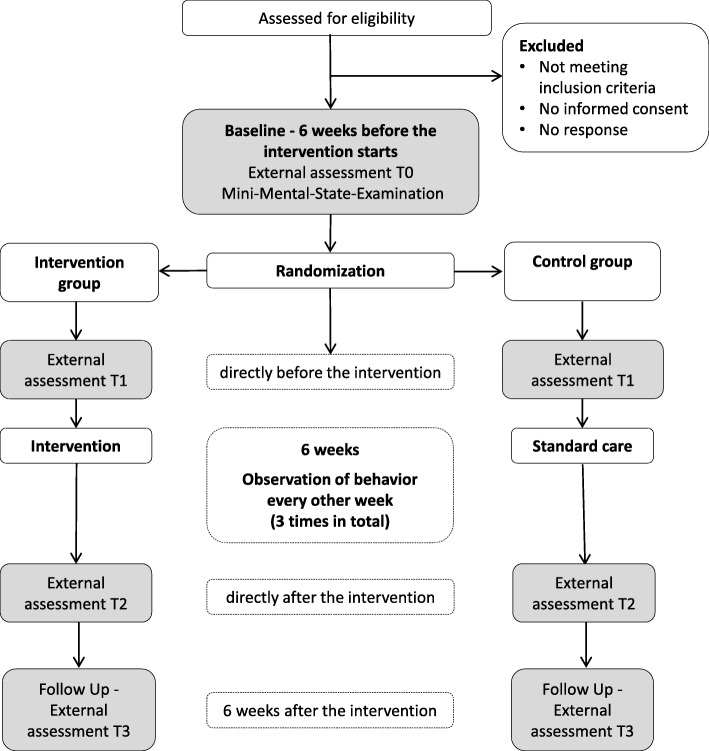
Trial Flow Diagram

At four assessment points, the nursing staff will conduct external assessments (see trial flow diagram, Fig. 1). Additionally, the participants’ behavior will be observed during the intervention period.

### Participants’ eligibility

The inclusion criteria for this study are the following: Participants have to be diagnosed with dementia (mild to severe) by a physician and live in institutional care. Participants (if they are able to) or legally authorized representatives (a relative or legal guardian) have to give written informed consent. Participants will be excluded if they have severe hearing problems.

### Recruitment

The study participants will be recruited in cooperation with the participating nursing homes in Thuringia. The nursing homes participating in the study are the following: Sophienhaus Weimar, Friedrich-Zimmer-Haus Weimar, Andreashof Erfurt and Martin-Luther-Haus Erfurt. If necessary to achieve the target sample size, a further nursing home will be recruited.

The nursing homes will send printed information about the study as well as a consent form for telephone contact with a member of the study team to relatives or legal guardians of potential study participants. If possible, a member of the project team will talk to potential participants themselves about the study and obtain their written consent to study participation. When PwD cannot give their consent themselves, the project staff will call relatives or legal guardians. After discussing open questions about the study with the relatives and legal guardians, the consent form will be sent to them with a stamped and addressed envelope.

### Ethical approval

Ethical approval was obtained from the Ethics Committee of the Faculty of Social and Behavioral Sciences of the Friedrich Schiller University Jena (committee’s reference number: FSV 18/06). Written informed consent will be obtained*.*

On the whole, the individualized music intervention represents a non-invasive and low risk intervention. As a possible risk of participating, agitated behavior may temporarily increase in response to the music selected for the individualized playlists. However, project staff or nursing home staff will monitor the participants and intervene if a negative reaction is observed during the music listening sessions. Songs to which participants repeatedly and specifically show negative reactions will be deleted from the playlist and replaced by other songs.

Participation in the study will be voluntary. Participants as well as their relatives or legal guardians can withdraw consent at any time.

### Primary outcome measures

At all four assessment points, quality of life and problem behavior will be assessed via external assessment by the nursing staff using different questionnaires.

#### Quality of life

Single items and visual analogue scales ranging from 0 to 100, based on Wilz and Soellner [29], will be used to assess emotional well-being, sleep quality, and social participation of the PwD. Depressive symptoms will be assessed with the Cornell Scale for Depression in Dementia (CSDD; [30]). The CSDD consists of 19 items and each item is rated on a scale of 0 to 2 (0 = absent, 1 = mild/intermittent, 2 = severe). Cronbach’s *α* is .84.

#### Problem behavior

To assess resistance to care via external assessment, a single item and visual analogue scale, based on Wilz and Soellner [29], will be used. In addition, agitated behavior will be assessed using the German version of the Cohen-Mansfield Agitation Inventory (CMAI; [31, 32]) which measures physically aggressive behaviors (PAB; e.g. hitting), physically non-aggressive behaviors (PNAB; e.g. general restlessness), and verbally agitated behaviors (VAB; e.g. repetitious sentences or questions) over the preceding two weeks. Each of the 29 items is rated on a seven-point scale of frequency. In addition, a five-point scale (from 1 = never to 5 = extremely) will be used for measuring disruptiveness of behavior. Cronbach’s *α* for the German version is .80 [32]. Furthermore, the German version of the Nurses Observation Scale for Geriatric Patients (NOSGER; [33]) will be used. It consists of six dimensions (memory, instrumental activities of daily living (IADL), basic activities of daily living (ADL), mood, social behavior, and disturbing behavior) with five items each. Each item is rated on a five-point scale of frequency of occurrence. The test-retest reliability for the six dimensions lies between .75 and .93.

In addition, goal attainment scaling (GAS; [34]) will be used to evaluate participants’ personal goal attainment. At T1 a member of the nursing staff will specify the participant’s personal goals for the individualized music intervention (IG) or for the standard offer of activities in the nursing home regarding the following six weeks (CG) in assistance by a member of the project team. At T2 nursing staff will rate participants’ personal goal attainment on a five-point scale, where point zero denotes the initial state being maintained or remaining stable. A lower value indicates a mild (− 1) or severe (− 2) deterioration from the certain goal, whereas positive scale values represent a positive change from the initial state in the sense of partial (+ 1) or complete (+ 2) goal attainment (cf. Chew et al. [35]).

During the intervention period, observation of behavior will be conducted three times for 60 min for each participant using an adapted rating scale (based on the Observed Emotion Rating Scale [36] and suggestions from the music&memory working group) with 37 items. The scale is divided into 15 time units of four minutes each. In each time unit it is coded whether the behavior occurs. The aim is to identify emotional and motor changes (facial expression, body movement, and other positive emotional reactions) and activation regarding social participation as well as the occurrence of problem behaviors or other negative reactions.

### Secondary outcome measures

Acceptance and applicability of the intervention will be measured via external assessment by the nursing staff at posttest (T2). For this purpose, a questionnaire (developed by the project team) was used which consists of one visual analogue scale measuring how helpful the music intervention was perceived for the PwD (0 “not helpful at all” to 100 “extremely helpful”) and the following five single items. The nursing staff will be asked to evaluate the frequency (too much, just right, too little) and duration (too long, just right, too short) of the music intervention as well as their satisfaction with the implementation (idea of an individualized music intervention and type of music: very dissatisfied, slightly dissatisfied, largely satisfied, or very satisfied), and whether they thought that the music sessions should be continued for each participant.

### Procedure

Six weeks before the intervention starts (T0), the severity of dementia will be assessed with the Mini-Mental State Examination (MMSE, [37]). At the same time, the nursing staff will conduct the first external assessments by completing different questionnaires regarding quality of life and problem behavior of PwD as described above. If the respective nursing staff member conducting the external assessment is uncertain about his or her assessment, a member of the project staff will attend the case conferences in the nursing home to obtain the missing information.

At T0, additionally relevant personal data of the participating PwD (e.g. medication, other physical and psychological diagnoses) will be assessed. As part of the assessments at T1, T2, and T3, any changes in personal data will be recorded. After completion of T0, participants will be randomly assigned to IG and CG.

Following randomization, the individualized playlists of the preferred music will be created for participants of the IG. We will gather information from family members, nursing home staff, and directly from participants if they are able to verbalize their personal preferences. A questionnaire developed by the project team (based on Gerdner [38], Gerdner and Schoenfelder [39], and a search of popular music from different decades) including a list of examples of popular artists and song titles will be used to identify personally-relevant music for each participant. Additionally, a telephone or face-to-face interview will be arranged with family members and/or participants themselves to get more detailed information about their music preferences. Based on this information, up to three individualized playlists for each participant will be compiled. Standardized documentation sheets will be used to record the characteristics of each playlist (number of playlist, number of tracks, duration of compilation).

Further external assessments by the nursing staff regarding quality of life and problem behavior will be conducted directly before (T1) and after the intervention (T2). At posttest, acceptance and applicability of the music intervention will be assessed. The follow-up assessment will take place six weeks after the end of the intervention (T3).

During the intervention period, the observations of behaviors will be conducted for IG and CG. To ensure a high methodological quality for the observations of behaviors, the project staff will be thoroughly trained in using the rating scale. At the beginning of the first intervention period, Cohen’s Kappa and percent agreement will be calculated to determine intercoder reliability. For this purpose, every observation of behavior will be conducted by two members of the project staff during the first two weeks. In case of an insufficient reliability, a training for improving intercoder agreement will be conducted.

Data will be collected on paper forms and data entry will be performed at the coordinating center in Jena by research assistants who are experienced in data entry. Double data entry will be performed for a random subset of data. All study-related information will be stored securely at the coordinating center.

### Intervention

Participants of the IG will listen to their individualized playlists on MP3 Players and headphones for 20 min every other day over the course of six weeks for a total of 21 music sessions. During the music intervention period, the project staff or nursing home staff will accompany and monitor the participants. As no comparable control intervention is available, the CG will receive standard care. Participants of both groups can use all simultaneous offers in the nursing home. If additional music therapy activities take place, they will be systematically documented. During the six weeks of intervention, observation of behavior will be conducted for the IG and the CG every other week for 60 min (three observations per participant). In the IG, participants’ behavior will be observed for 20 min before the music listening session starts, during the 20-min listening session, and for 20 min after the end of the music listening session.

For all music listening sessions, date, time, and duration of the session as well as, if necessary, the reason why a session had to be cancelled will be recorded by project staff or staff from the nursing home using a standardized documentation sheet. The suitability of the playlists will be checked and documented during the music listening sessions and playlists will be continuously adapted over the intervention period as needed.

### Sample size

Based on findings from a meta-analysis [27], small effect sizes on quality of life and problem behavior of PwD are expected (*f* = 0.2).

Hypothesizing this small effect size in a two-group design, an *α* of .05 and a power (1 − β) of 0.95, a sample of *n* = 92 participants is needed. Assuming a dropout rate of 27% (18% [40] and 36% [21]), 130 PwD will be recruited.

### Monitoring

A data monitoring committee will not be necessary, as the clinical trial does not involve a high-risk intervention. We do not expect adverse events or other unintended effects of the intervention. During the entire study period, participants will be accompanied by the project staff and nursing home staff.

### Data analysis

The data will be analyzed using SPSS (IBM Corp., Armonk, NY) and R (R Development Core Team, Vienna, Austria).

Descriptive statistics of playlist characteristics, number of music sessions, music session duration, the proportion of cancelled sessions, and why sessions were cancelled will be reported as indicators of implementation quality.

To check whether the randomization was successful, baseline characteristics and the pre-test levels of the outcome measures will be compared between IG and CG. Further analyses will be adjusted for any potential significant differences.

“As randomized” analyses will be performed retaining participants in the group to which they were originally allocated regardless of protocol adherence. In addition, per protocol analyses will be conducted based on the sample of participants who adequately adhered to the intervention protocol by completing at least five out of the 21 music listening sessions. Any differences between protocol non-adherers and protocol adherers as well as dropouts and those who complete the study will be analyzed regarding baseline characteristics and pre-test levels of the outcome measures.

Multilevel models for discontinuous change [41] will be performed for the primary outcomes to evaluate whether outcomes show different change patterns between IG and CG from baseline (T0) to pretest (T1) to posttest (T2) to follow-up (T3). Following Göllner et al. [42], three contrast variables will be computed by subtracting the baseline values from pretest (T1-T0), posttest (T2-T0) and follow-up values (T3-T0), respectively. The multilevel models will have two levels: the four assessments of the outcome variables as level-1 and participants as level-2. To examine average treatment effects, group membership will be included in the multilevel models as a dichotomous level-2 predictor (0 = CG, 1 = IG). Differential effectiveness will be analyzed by including the severity of dementia and its interaction term with the group membership variable as person-level moderator variables. Full maximum likelihood estimation will be used to account for missing data.

Average personal goal attainment according to the GAS-ratings of the nursing staff will be calculated by first calculating the average personal goal attainment for each participant across all of his/her specified goals, and then calculating the average goal attainment across all participants separately for IG and CG. In addition, the proportion of participants who completely or partially attained their goals, experienced at best no change with regard to their personal goals, and the proportion who reported mild or severe deterioration on the specified goals separately for IG and CG will also be calculated. Frequency scores will be compared between the two groups.

For analyzing the behavioral observation data, frequency scores will be computed for time units 1–5 (i.e. the first 20 min of behavior observation; pre-intervention period in the IG), 6–10 (i.e. the second 20-min period; intervention period in the IG) and 11–15 (i.e. the third 20-min period; post-intervention period in the IG) by summing up how often each behavior occurred in each of the three observation periods. Frequency scores for each period will subsequently be compared both within and between intervention groups in order to identify significant changes in frequencies of behaviors.

Frequencies and percentages in each response category will be computed for the secondary outcome measures as indicators of the acceptance and applicability of the intervention.

### Dissemination

All results of the study will be published in international and open-access journals. In addition, the results will be presented at meetings (e.g. in the participating nursing homes) and congresses.

## Discussion

This study will evaluate whether an individualized music intervention can be successfully implemented in the institutional care setting and improve the quality of life of PwD living in a nursing home.

The study has several strengths. To address the methodological limitations of previous studies, a RCT with a large sample of PwD living in multiple nursing homes is conducted. Our study also includes the systematic identification of personally-relevant music for each participant, the continuous examination and adaptation of playlists, and a higher dosage of individualized music listening sessions in comparison with the interventions evaluated in previous studies. We also address limitations of previous studies by following a careful implementation procedure and by evaluating the implementation success.

Another strength of this study is its robust design with four assessment points including a baseline, pretest, posttest, and follow-up assessment allowing the evaluation of different change patterns between IG and CG, as well as its long-term effects of the individualized music intervention. The study design also allows for controlling the progression of dementia. Additionally, the multiple, detailed and controlled observation of behavior constitutes an innovative novelty in the context of individualized music interventions. PwD with all severity stages will be included in the study.

However, there are also limitations identified in this study. First, nearly all outcomes will depend on the external assessment by the nursing staff. Nevertheless, the outcomes will be disease specific and, in our opinion, assessments by nursing staff will be most valid for this purpose. In addition, observation of behavior will be performed due to trained raters. Future research may also include physiological outcomes (e.g. cortisol, heart rate) as potential outcome measures.

Second, blinding is not possible in the design of the proposed study since the nursing home staff and project staff will accompany and monitor the participants during the intervention period and will, therefore, know which participants receive the music intervention and which do not.

On the whole, individualized music represents a promising non-invasive and low-risk intervention that can be delivered easily without a professional in contrast to active forms of music therapy (i.e. singing or moving to music). Thus, the individualized music intervention may be well integrated in the care setting and our results regarding the acceptability as well as the implementation will provide insights into this.

In summary, this will be the first study to carefully test the implementation, efficacy, and acceptability of an individualized music intervention for PwD in multiple nursing homes in Germany within a RCT including a large sample. If the individualized music intervention proves to be effective in improving primary outcomes, our results will be relevant to practice. Hence, individualized music interventions could be used as an innovative and inexpensive alternative to pharmacological interventions for PwD in the institutional care setting. Therefore, the results of the study will be used to improve the quality of life of PwD living in a nursing home.

## Abbreviations

CG: Control group
GAS: Goal attainment scaling
IG: Intervention group
PwD: People with dementia
RCT: Randomized controlled trial
T0: Baseline assessment
T1: Pretest assessment
T2: Posttest assessment
T3: Follow-up
